# Impact of Boron Acceptors on the TADF Properties of *Ortho*-Donor-Appended Triarylboron Emitters

**DOI:** 10.3389/fchem.2020.00538

**Published:** 2020-06-24

**Authors:** Hanif Mubarok, Woochan Lee, Taehwan Lee, Jaehoon Jung, Seunghyup Yoo, Min Hyung Lee

**Affiliations:** ^1^Department of Chemistry, University of Ulsan, Ulsan, South Korea; ^2^School of Electrical Engineering, KAIST, Daejeon, South Korea

**Keywords:** TADF, *ortho*-donor-acceptor, triarylboron, cyclic boryl acceptor, OLEDs

## Abstract

We report the impact of boron acceptors on the thermally activated delayed fluorescence (TADF) properties of *ortho*-donor-appended triarylboron compounds. Different boryl acceptor moieties, such as 9-boraanthryl (**1**), 10*H*-phenoxaboryl (**2**), and dimesitylboryl (BMes_2_, **3**) groups have been introduced into an *ortho* donor (D)–acceptor (A) backbone structure containing a 9,9-diphenylacridine (DPAC) donor. X-ray crystal diffraction and NMR spectroscopy evidence the presence of steric congestion around the boron atom along with a highly twisted D–A structure. A short contact of 2.906 Å between the *N* and *B* atoms, which is indicative of an N → B nonbonding electronic interaction, is observed in the crystal structure of **2**. All compounds are highly emissive (PLQYs = 90–99%) and display strong TADF properties in both solution and solid state. The fluorescence bands of cyclic boryl-containing **1** and **2** are substantially blue-shifted compared to that of BMes_2_-containing **3**. In particular, the PL emission bandwidths of **1** and **2** are narrower than that of **3**. High-efficiency TADF-OLEDs are realized using **1**–**3** as emitters. Among them, the devices based on the cyclic boryl emitters exhibit pure blue electroluminescence (EL) and narrower EL bands than the device with **3**. Furthermore, the device fabricated with emitter **1** achieves a high external quantum efficiency of 25.8%.

**Graphical Abstract:**
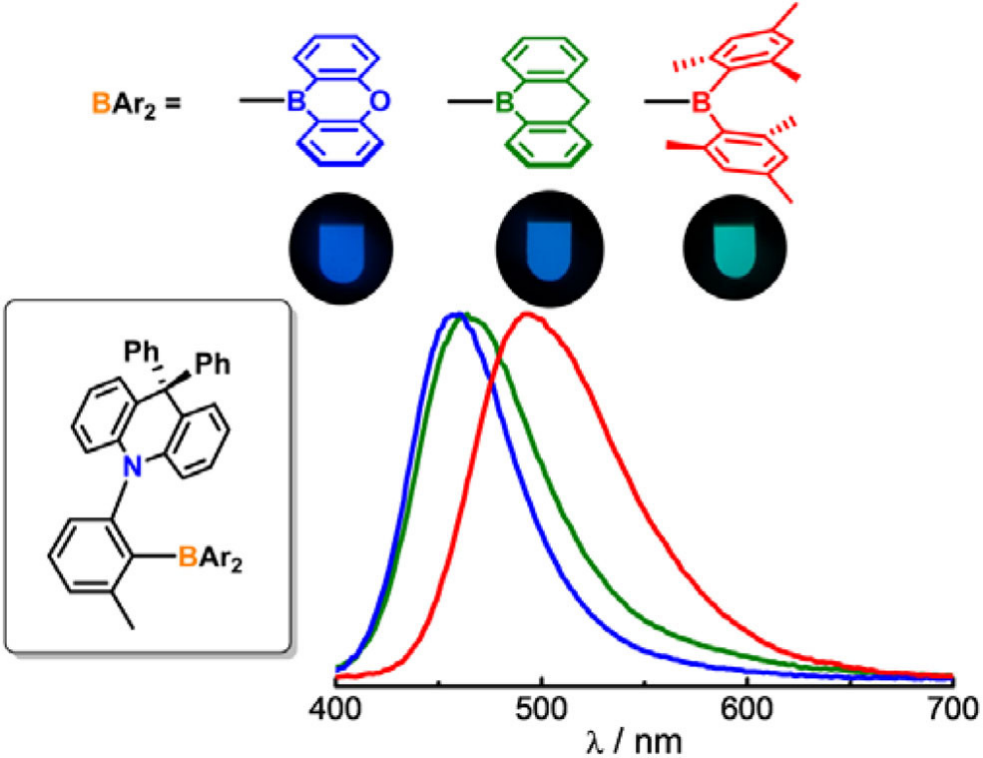
Impact of boron acceptors on the TADF properties of *ortho*-donor-appended triarylboron emitters.

## Introduction

Thermally activated delayed fluorescence (TADF) compounds have recently received great attention as efficient emitters in organic light-emitting diodes (OLEDs) because TADF-OLEDs can theoretically achieve nearly 100% internal quantum efficiency (η_int_) via the upconversion of triplet excitons into emissive singlet excitons through a thermally activated reverse intersystem crossing (RISC) process (Goushi et al., 2012; Uoyama et al., 2012; Zhang et al., 2012, 2014; Dias et al., 2013; Tao et al., 2014; Hirata et al., 2015; Kaji et al., 2015; Im et al., 2017; Wong and Zysman-Colman, 2017; Yang et al., 2017; Cai and Su, 2018; Kim et al., 2018). To date, various types of TADF emitters have been reported, and those containing boron acceptor moieties are recently attracting special interest because of their excellent TADF properties. Boron acceptors such as triarylborons possess strong electron-accepting properties owing to their sp^2^ hybridized, tri-coordinate boron atom, which has a vacant p(B) orbital (Hirai et al., 2015; Hatakeyama et al., 2016; Turkoglu et al., 2017; Matsui et al., 2018). In addition to this electron-deficiency, p(B)–π^*^ conjugation between the boron atom and linked π systems can lead to the stabilization of the LUMO level. Thus, in combination with suitable donors, the boron acceptors may form donor–acceptor (D–A) emitters exhibiting TADF. In fact, boron-based emitters have been successfully employed in blue and green OLEDs (Kitamoto et al., 2015, 2016; Numata et al., 2015; Suzuki et al., 2015; Liu et al., 2016; Lien et al., 2017; Chen et al., 2018a,b; Liang et al., 2018; Tsai et al., 2018; Meng et al., 2019; Wu et al., 2019). For example, TADF-OLEDs based on diboron and oxaborin emitters show excellent device performance with a very high external quantum efficiency (EQE) of over 38% (Wu et al., 2018; Ahn et al., 2019). Hatakeyama et al. recently demonstrated that boron and nitrogen-doped polycyclic aromatic hydrocarbons exhibit a deep-blue emission with a very narrow band (full width at half maximum, FWHM = 18 nm), and achieve a high EQE of 34% when incorporated in OLEDs (Kondo et al., 2019). Our group also reported that *ortho*-donor-appended triarylboron compounds have strong TADF character because of their highly twisted D–A structure (Lee et al., 2017). We have shown that electronic modification of the donor and/or boryl acceptor moiety in the *ortho* compounds allows facile tuning of the HOMO and LUMO levels, which in turn tunes the emission color over the visible region (Kumar et al., 2019). In particular, TADF-OLEDs having *ortho* compounds as emitters display a high EQE of above 30% in blue OLED devices (Lee et al., 2018). These results demonstrate that boron compounds having an *ortho*-D–A scaffold constitute highly efficient TADF emitters due to their rigid backbone structure.

Considering the growing interest in developing new boron-based emitters for enhancing the efficiency and stability of devices thereof and reducing the emission bandwidth, it is important to systematically examine the effects of boron acceptors on the TADF properties of the emitters. As noted in previous reports, the most widely adopted boron acceptors are dimesitylboryl (BMes_2_) and heteroatom-bridged cyclic boryl moieties (Hirai et al., 2015; Kitamoto et al., 2015; Liang et al., 2018; Park et al., 2018; Meng et al., 2019; Wu et al., 2019). While the former is effective in providing a steric effect due to its bulky nature, the latter is beneficial for attaining a rigid structure with adjustable electronic effects (Hirai et al., 2019). To elucidate the impact of the boron acceptors on the TADF properties in more detail, we set out to explore a series of *ortho*-donor-appended triarylboron compounds (**1**–**3**), which contain different boryl acceptor moieties such as BMes_2_ and cyclic boryl groups and a fixed donor. The photophysical properties of these boron-based emitters are examined along with theoretical consideration. We demonstrate that the performance of TADF-OLEDs fabricated with these emitters varies with the type of acceptor, and a high EQE of over 25.8% is realized in pure blue devices with the emitter containing a cyclic boryl acceptor.

## Results and Discussion

### Synthesis and Characterization

**Scheme 1:**
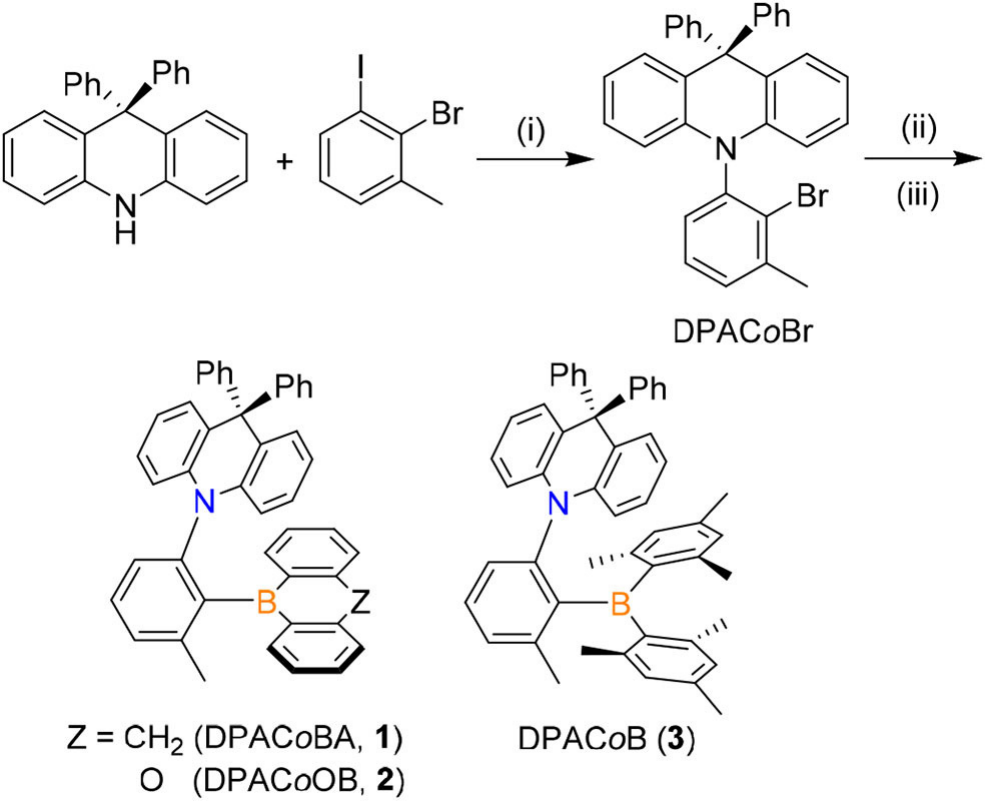
Synthesis of *ortho*-DPAC-appended triarylboron compounds (**1**–**3**). Conditions: (i) Pd_2_(dba)_3_, P(^*t*^Bu)_3_, NaO^*t*^Bu, toluene, 110°C. (ii) *n*-BuLi, ether, −30°C. (iii) Ar_2_BX (9-bromo-9,10-dihydro-9-boraanthracene for **1**; 10-bromo-9-oxa-10-boraanthracene for **2**; dimesitylboron fluoride for **3**), toluene, −78°C.

Triarylboron compounds (**1**–**3**) in which a 9,9-diphenylacridine (DPAC) donor (D) is linked to a boryl acceptor (A) in the *ortho* position of the phenylene ring were prepared (Scheme 1). The cyclic boryl groups [9-boraanthryl (**1**) and 10*H*-phenoxaboryl (**2**)] and a dimesitylboryl group (BMes_2_, **3**) were introduced as acceptors. Buchwald–Hartwig amination reactions between 9,9-diphenyl-10*H*-acridine and 2-bromo-1-iodo-3-methylbenzene produced an *ortho*-DPAC-substituted bromobenzene intermediate, DPAC*o*Br. The lithium salt of DPACoBr was then subjected to reaction with the corresponding boryl-halides to afford the final *ortho*-DPAC-appended triarylboron compounds **1**–**3**. As expected, compound **3** bearing a bulky BMes_2_ group was very stable in air and water. In addition, compounds **1** and **2** having cyclic boryl groups were stable under ambient conditions, presumably due to the steric protection of the boron center by the *ortho*-DPAC and -Me groups. Consequently, all compounds exhibited high thermal stability, as judged by their high decomposition temperatures (*T*_d5_) over 320°C.

The compounds were characterized by multinuclear NMR spectroscopy, elemental analysis, and single crystal X-ray crystallography. The ^1^H NMR spectra of **1** and **2** exhibit sharp signals for the cyclic boryl groups, whereas all the methyl and C_Mes_-H protons on the two Mes groups in **3** give rise to separate singlets. This feature indicates the highly restricted motion of the bulky BMes_2_ moiety of **3** in solution, which can be attributed to the severe steric hindrance from the *ortho*-DPAC and -Me groups on the phenylene ring (Supplementary Figures 2–4) (Lee et al., 2017, 2018). In particular, while the ^11^B NMR spectrum of **3** shows a broad signal at δ 84 ppm, typical of base-free, triarylboron compounds (Wade et al., 2010; Lee et al., 2017), the ^11^B signals of **1** and **2** are observed at more upfield regions (δ 58 ppm for **1** and δ 65 ppm for **2**). This can be mainly ascribed to the π-donation effect from the ipso-carbon atoms to the empty p(B) orbital due to the planar structure of the cyclic boryl moieties in **1** and **2** (Zhou et al., 2012).

**Figure 1:**
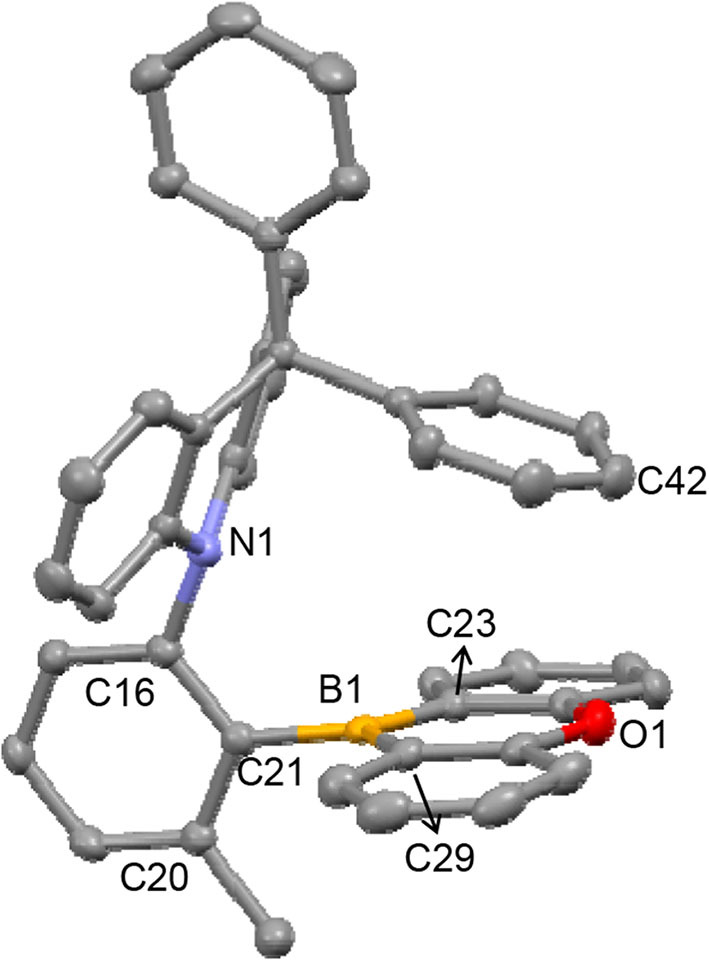
X-ray crystal structure of **2** (40% thermal ellipsoids). The H-atoms are omitted for clarity. Selected bond lengths (Å) and angles (°): B1–C21 = 1.580(2), B1–C23 = 1.532(2), B1–C29 = 1.528(2); ∠OB–Ph = 83.7, ∠DPAC–Ph = 86.4, N1–C16–C21 = 117.99(13), C16–C21–B1 = 121.68(13), Σ(C–B–C) = 359.7; Interatomic distances (Å): N1···B1 = 2.906; C42···O1 = 3.462.

X-ray diffraction studies conducted on DPAC*o*OB (**2**) confirmed that it exhibits an *ortho* D–A structure, in which both the DPAC and OB rings are almost orthogonal to the phenylene ring (∠DPAC–Ph = 86.4° and ∠OB–Ph = 83.7°), thus facing each other (Figure 1). The distance between the N1 and B1 atoms is short (2.906 Å), lying within the sum of the van der Waals radii of the two atoms. This may indicate the presence of an N → B nonbonding electronic interaction. Interestingly, the DPAC ring is puckered at the 9-position, because of the sp^3^ character of the 9-carbon atom, in such a way that one peripheral Ph ring protrudes right above the OB ring. Although the nearest contact between the Ph and OB rings (C42···O1) is relatively long (3.462 Å), this feature might indicate the occurrence of a π-π interaction between the Ph and OB planes. These structural aspects suggest that the boron atom in **2** is sterically and electronically protected, which contributes to its chemical and thermal stability. In the boryl moiety, the boron atom possesses a trigonal planar geometry with a Σ(C–B–C) of 359.7°. It is noteworthy that the two B–C(OB) bond lengths (1.532 and 1.528 Å) are much shorter than that of the B–C(Ph) bond (1.580 Å), the latter being within the range usually found for triarylborons such as Ph_3_B (Zettler et al., 1974) and Mes_3_B (1.57–1.59 Å) (Blount et al., 1973). As similarly noted in the upfield shifts of the ^11^B signals, this finding can be attributed to the presence of p(B)–π interactions in the oxaborin ring, which shorten the B–C(OB) bond lengths.

### Photophysical and Electrochemical Properties

**Figure 2:**
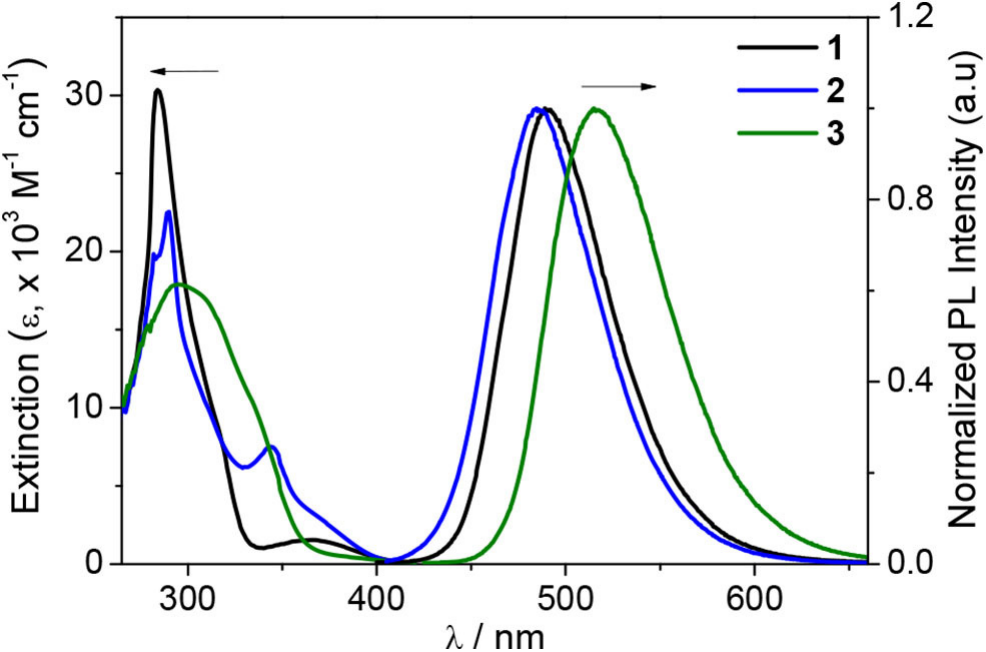
UV/vis absorption (left) and PL spectra (right) of **1**–**3** in toluene (2.0 × 10^−5^ M) at RT.

The photophysical properties of the compounds were investigated by UV/vis absorption and photoluminescence (PL) measurements in toluene (Figure 2 and Table 1). The intense high-energy absorptions observed at ca. 290 nm can be mainly attributed to the local π-π^*^ transitions centered in the DPAC donor (Park et al., 2018), and the broad bands or shoulders ranging from ca. 300 to 350 nm are assignable to the boryl centered π-π^*^ transitions with π(Ar)–p(B) CT character (see the DFT results below) (Hudson and Wang, 2009; Wade et al., 2010; Zhou et al., 2012). The weak low-energy band for **1** or the tailing found for **2** and **3** above ca. 350 nm can be ascribed to the intramolecular CT (ICT) transitions from the DPAC donor to the boryl acceptor moieties.

**Table 1:** Photophysical data of *ortho*-donor-appended triarylboron compounds (**1–3**) in toluene.

**Compd**	**$\lambda_{\text{abs}}^{\text{a}}$** **(nm)**	**$\lambda_{\text{PL}}^{\text{a}}$** **(nm)**	**$\lambda_{\text{FWHM}}^{\text{b}}$** **(nm)**	**$\phi_{\text{PL}}^{\text{a,c}}$** **N_**2**_/air (%)**	**$\tau_{\text{p}}^{\text{a,d}}(\phi_{\text{PF}})$** **[ns (%)]**	**$\tau_{\text{d}}^{\text{a,d}}(\phi_{\text{DF}})$** **[μs (%)]**	**HOMO/LUMO^**e**^** **(eV)**	**$E_{\text{g}}^{\text{f}}$** **(eV)**	**$\Delta E_{\text{ST}}^{\text{g}}$** **exp/calc (eV)**
**1** (DPAC*o*BA)	284, 318(sh), 367	490	64	91/5.9	62.2 (36)	8.1 (55)	−5.31/−2.61	2.70	0.006/0.039
**2** (DPAC*o*OB)	290, 344, 368(sh)	485	66	99/6.1	173.4 (52)	8.5 (47)	−5.34/−2.50	2.84	0.020/0.048
**3** (DPAC*o*B)	295, 335(sh)	516	73	99/4.6	200.7 (13)	8.3 (86)	−5.34/−2.52	2.82	0.013/0.051

a *In oxygen-free (N_2_) toluene at 298 K (5.0 × 10^-5^ M)*.

b *Full width at half maximum (FWHM)*.

c *Absolute PLQYs*.

d *PL lifetimes of prompt (τ_p_) and delayed (τ_d_) decay components. The prompt (Φ_PF_) and delayed (Φ_DF_) portions (%) are given in parentheses*.

e *Estimated from the electrochemical oxidation (HOMO) and reduction (LUMO)*.

f *Electrochemical band gap*.

g *ΔE_ST_ = E_S_ – E_T_. Singlet (E_S_) and triplet (E_T_) energies from the fluorescence and phosphorescence spectra at 77 K. Calculated ΔE_ST_ from TD-DFT at PBE0/6-31G(d,p)*.

**Figure 3:**
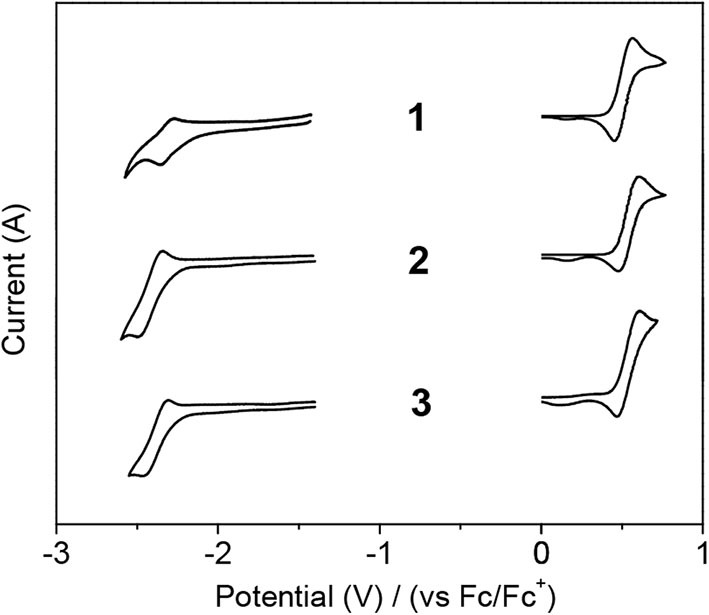
Cyclic voltammograms of **1**–**3** in solution (1 × 10^−3^ M) showing reduction (left) and oxidation (right). Solvent: CH_2_Cl_2_ for oxidation and DMSO (**1**) or THF (**2** and **3**) for reduction.

To elucidate the ICT transition, the electrochemical properties of the compounds were investigated by cyclic voltammetry (Figure 3 and Table 1, Supplementary Table 2). All compounds exhibit DPAC-centered oxidation with similar potential values (*E*_ox_ = 0.51–0.54 V) probably because they have the same donor moiety and orthogonal D–A arrangement. However, minor peaks are concomitantly observed at ca. 0.15 V, which might originate from side reactions, such as possible dimerization of radical cations derived from DPAC moieties. As for the reduction, all compounds undergo similar boron-centered reduction with slightly different reduction potentials. Unlike usual triarylboron-based reductions, the reduction process is not completely reversible, but rather to be quasi-reversible. Oxaborin-containing **2** shows the most negative value presumably due to an electron donating effect of oxygen lone pairs through the planar structure, which raises the LUMO level. Although the reduction of boraanthracene-containing **1** occurs at a more positive position, it was measured in DMSO instead of THF because the reduction of **1** was unclear in the latter solvent. It seems that the DMSO solvent stabilizes the boryl moiety, resulting in a more facile reduction. Hence, the band gap (*E*_g_) of **1** is smaller than that of **2** despite the similar ICT absorption wavelength of both compounds in toluene (Table 1).

**Figure 4:**
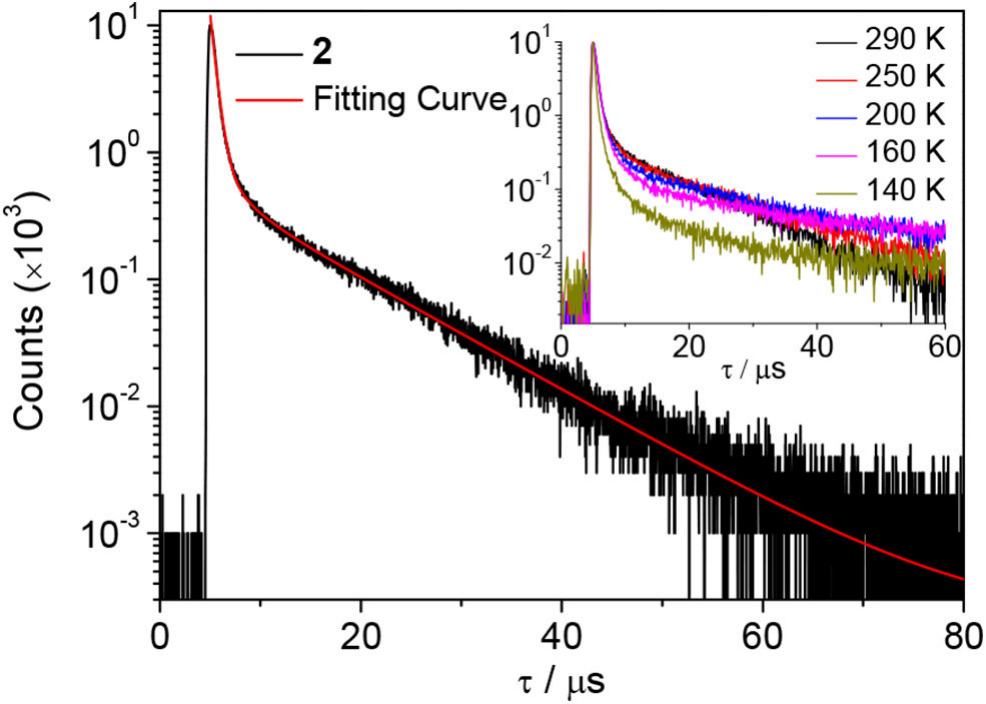
Transient PL decay curve of **2** in oxygen-free toluene at 298 K. Inset: temperature-dependent PL decay.

Next, the emission properties of all compounds were examined in toluene (Figure 2). The PL spectra show broad emission bands typical for an ICT transition. Compounds **1** and **2** exhibit sky blue emissions at similar wavelengths (λ_PL_ = 490 nm for **1** and 485 nm for **2**), whereas **3** displays green emission at 516 nm. In particular, the PL emission bandwidths of **1** and **2** with cyclic boryl moieties (λ_FWHM_ = 64–66 nm) are narrower than that of **3** with a BMes_2_ acceptor (λ_FWHM_ = 73 nm). This indicates a small structural deformation between the ground and excited states of **1** and **2** in solution, presumably due to the rigid cyclic boryl groups. All compounds are highly emissive in oxygen-free toluene with high PL quantum yields (PLQY, Φ_PL_) of over 90%; the PLQYs of **2** and **3** are close to 99%, and that of **1** is ca. 91%. In sharp contrast, the PLQYs in air-saturated toluene show drastically decreased values of ca. 5–6% (Table 1 and Supplementary Figure 6). This result suggests that efficient T_1_ to S_1_ RISC takes place in oxygen-free toluene, thus pointing to the occurrence of strong delayed fluorescence. In fact, the transient PL decay curves exhibit intense delayed components with microsecond lifetimes (τ_d_) along with prompt components (Figure 4 and Supplementary Figure 6). The temperature-dependent PL decay also confirms that the delayed component is assignable to TADF (inset in Figure 4) (Uoyama et al., 2012). The Δ*E*_ST_ values are very small, below 0.02 eV, supporting a fast equilibration between the S_1_ and T_1_ states. The delayed fluorescence lifetimes (τ_d_) of **1**–**3** are very similar to each other (ca. 8 μs) and correlate with the Δ*E*_ST_ values. However, the delayed portion (Φ_DF_) in the transient decay curve is different depending on the boryl acceptors. BMes_2_-containing **3** has the largest portion of delayed fluorescence (Φ_DF_ = 86%), whereas the Φ_DF_ values for compounds **2** and **3** having cyclic boryl acceptors are in the range of ca. 47–55%.

The photophysical properties of the compounds were further investigated in a doped host film (DPEPO, bis[2-(diphenylphosphino)phenyl]ether oxide) containing 20 wt% of compounds (Table 2 and Supplementary Figure 8). Although the PL wavelengths show the same trend as that found in solution, all compounds display substantial rigidochromic blue-shifts by ca. 21–34 nm. We attribute this result primarily to the high rigidity of **1**–**3** in film state due to the steric effects of the *ortho*-DPAC and -Me groups. Moreover, the shifts observed for compounds **1** and **2** are greater than those of **3**. This is most likely due to the additional rigidity endowed by the cyclic boryl moieties. As similarly found in solution, the PL emission of **1** and **2** is slightly narrower than that of **3** in the film state (λ_FWHM_ = 62–71 nm for **1** and **2** vs. 74 nm for **3**). In particular, the PLQYs in the host film (Φ_PL_ = 90–97%) are very high and comparable to those obtained in solution, also with the value for **1** being lower than those for **2** and **3**. The delayed fluorescence having long lifetimes (ca. 6.5–8.8 μs) and large portions of the delayed components indicate that the strong TADF character of **1**–**3** is well-retained in film state. It is noteworthy that the portion of the delayed fluorescence follows the order **3** > **1** > **2**, as identically observed in solution state.

**Table 2:** Photophysical data of DPEPO films doped with 20 wt% of **1–3**.

**Compd**	**λ_**PL**_ (nm)**	**$\Delta \lambda_{\text{PL}}^{\text{a}}$ (nm)**	**$\lambda_{\text{FWHM}}^{\text{b}}$ (nm)**	**Φ_**PL**_ (%)**	**Φ_**PF**_ (%)^**c**^**	**Φ_**DF**_ (%)^**c**^**	**τ_**d**_ (μs)^**d**^**
**1**	463	27	71	90	34	56	8.8
**2**	451	34	62	97	44	53	6.5
**3**	495	21	74	96	15	81	8.0

a *Difference in the PL wavelengths obtained in toluene solution and DPEPO film*.

b *Full width at half maximum (FWHM)*.

c *Prompt (Φ_PF_) and delayed (Φ_DF_) portions (%) in transient PL decay curves*.

d *PL lifetimes of delayed decay components*.

### Theoretical Calculations

**Figure 5:**
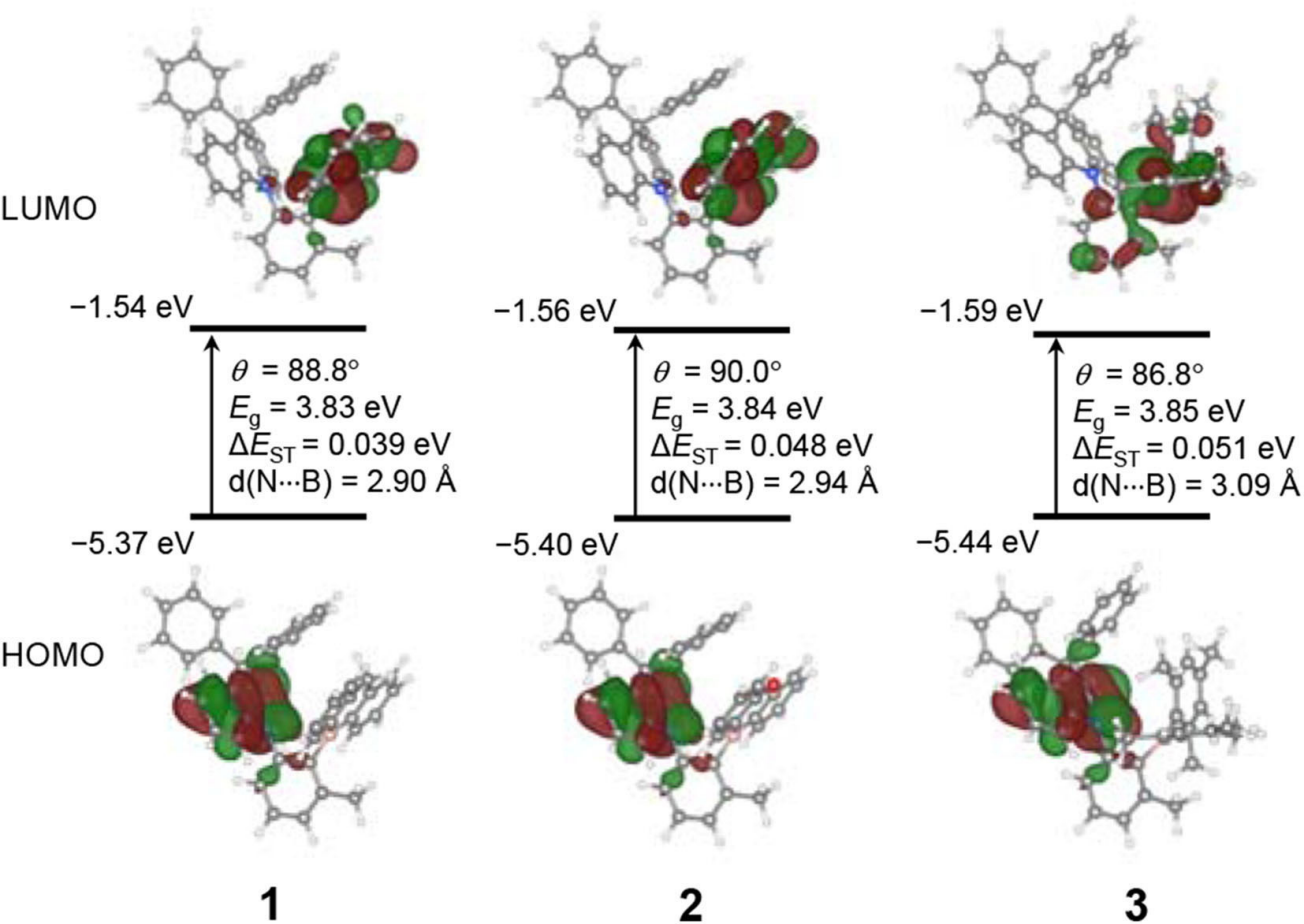
Frontier molecular orbitals, HOMO and LUMO, of **1**–**3** (isovalue = 0.03) at their ground state (S_0_) geometries from PBE0/6-31G(d,p) calculations. MO energies, dihedral angles (∠DPAC–Ph, θ), HOMO–LUMO gaps (*E*_g_), energy splitting between the S_1_ and T_1_ states (Δ*E*_ST_), and N···B interatomic distances are provided.

To gain a deeper understanding of the geometric structures and photophysical properties of compounds **1–3**, computational studies based on density functional theory (DFT) were performed at the PBE0/6-31G(d,p) level. Optimization of the ground state (S_0_) and excited state (S_1_ and T_1_) geometries was made by DFT and time-dependent DFT (TD-DFT) methods, respectively (Figure 5 and Supplementary Table 3). The short N···B contacts (ca. 2.90–2.94 Å) in **1** and **2** are comparable to that found in the crystal structure of **2** (2.906 Å), whereas that in **3** shows an increased distance of 3.09 Å. All compounds exhibit high dihedral angles close to 90°, which are also comparable to the experimental value for **2** (86.4°), between the DPAC donor and the phenylene ring at the ground state. As a result, the HOMOs and LUMOs are spatially separated and located on the DPAC donor and boryl acceptor moieties, respectively. In particular, the LUMOs of **1** and **2** are almost exclusively contributed by the cyclic boryl moieties, whereas **3** has a substantial LUMO contribution from the phenylene ring (ca. 19%). This finding can be mainly attributed to the strong p(B)–π^*^ electronic conjugation in the cyclic boryl moieties. Moreover, unlike the propeller-like conformation of the PhBMes_2_ moiety in **3**, the cyclic boryl rings in **1** and **2** are nearly orthogonal to the phenylene ring. This may weaken the LUMO conjugation between the two rings, which results in the cyclic boryl moieties dominating the LUMOs. The resulting LUMO level is slightly lowered for **3** compared to those of **1** and **2**, as shown in the electrochemical reduction. The computed Δ*E*_ST_ values are in the range of ca. 0.04–0.05 eV for all compounds, similarly to the experimental values. The very small Δ*E*_ST_ values support the observed strong TADF properties. Although the HOMO–LUMO band gaps of all compounds in the ground state are very similar, the S_1_ state energy and TD-DFT calculation predict that the lowest-energy absorption and emission energies follow the order **2** > **1** > **3**, which is in agreement with the experimental results and corroborates the blue-shifted emission of cyclic boryl-containing **1** and **2** (Supplementary Tables 3–5).

### Electroluminescent Properties

**Figure 6:**
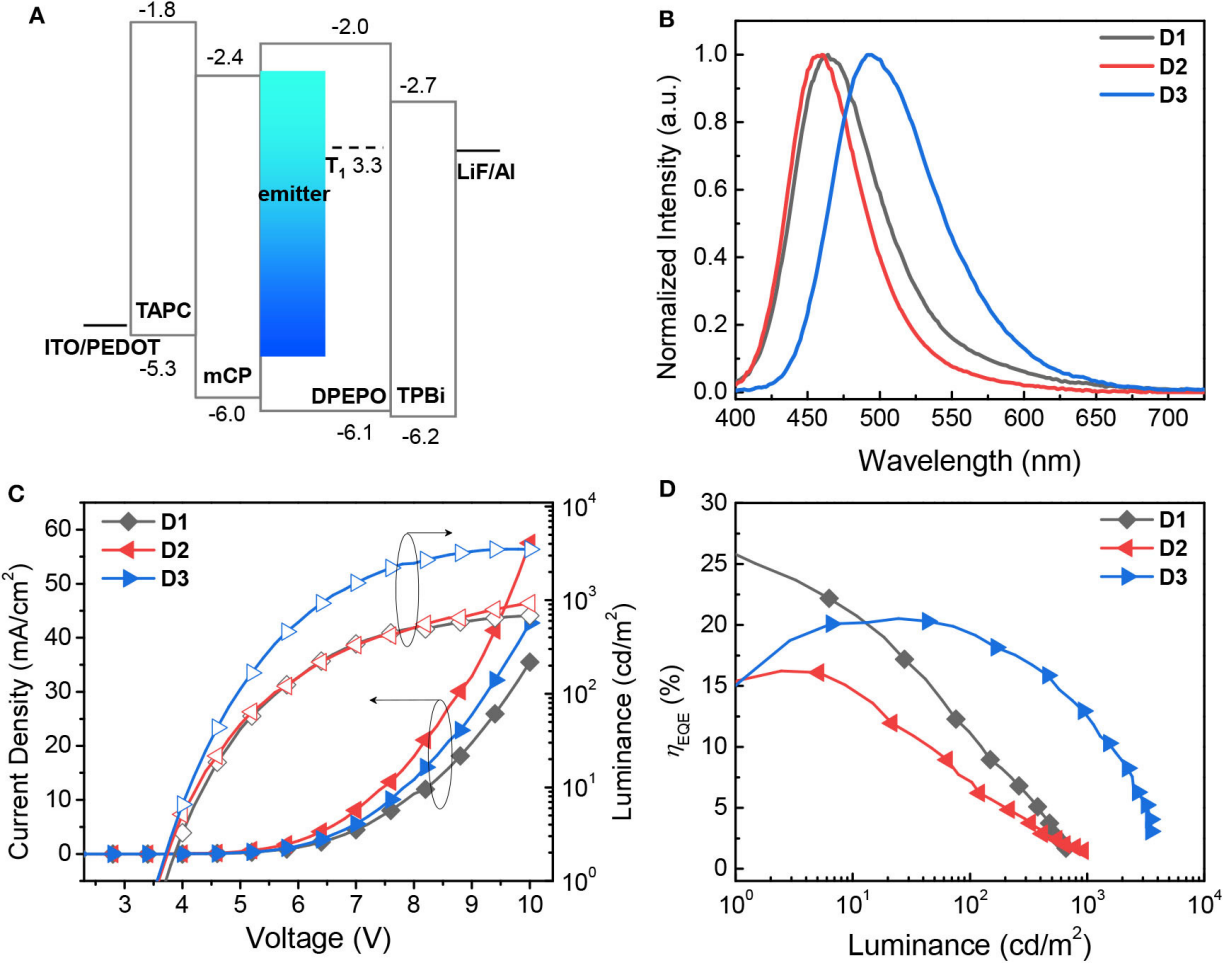
**(A)** Energy level diagram of devices (in eV) relative to the vacuum level. **(B)** EL spectra of devices **D1–D3** with the proposed TADF emitters. **(C)** Current density–voltage–luminance (*J*–*V*–*L*) characteristics of the **D1–D3** devices. **(D)** External quantum efficiency–luminance (η_EQE_-*L*) characteristics of the **D1–D3** devices.

To investigate the electroluminescent (EL) properties of the proposed emitters **1**–**3**, TADF-OLED devices having the following structure were fabricated (Figure 6A): glass/indium-tin-oxide (ITO) (150 nm)/poly(3,4-ethylenedioxy-thiophene):poly(styrenesulfonate) (PEDOT:PSS) (40 nm)/1,1-bis[(di-4-tolylamino)phenyl]cyclohexane (TAPC) (20 nm)/1,3-bis(*N*-carbazolyl)benzene (mCP) (10 nm)/bis[2-(diphenylphosphino)phenyl]ether oxide (DPEPO):emitter (20 wt%, 25 nm)/DPEPO (10 nm)/2,2′,2"-(1,3,5-benzinetriyl)-tris(1-phenyl-1-*H*-benzimidazole) (TPBi) (30 nm)/lithium fluoride (LiF) (1 nm)/aluminum (Al) (100 nm) (**D1**–**D3**). Because of their blue emitting properties, the emitters were doped into a DPEPO host with a high T_1_ energy (T_1_ = 3.3 eV). Similarly to previous pure blue devices (Lee et al., 2017, 2018), the emitters were codeposited with the host at a dopant concentration of 20 wt%. The preliminary results for the device performance are summarized in Table 3. The EL emission of the devices with the three different dopants varies from pure blue [CIE (x, y) = (0.151, 0.128)] to cyan [CIE (x, y) = (0.208, 0.421)], which is similar to the PL spectra of the host films doped with the corresponding emitters (Figure 6B). All the devices show negligible spectral variations over the whole angle range, which is indicative of their wide angular stability (Supplementary Figure 10). In particular, as consistently observed in the PL spectra of the emitters, the EL emissions of **D1** and **D2** having the emitters based on the cyclic boryl acceptors are narrower than that of **D3** (λ_FWHM_ = 59–70 nm for **D1** and **D2** vs. 83 nm for **D3**). According to the current density–voltage–luminance (*J*–*V*–*L*) characteristics and the external quantum efficiency–luminance (EQE–*L*) characteristics of the devices shown in Figures 6C,D, all the devices exhibit very good performance with high EQE values. In fact, device **D1** achieves a very high maximum EQE of 25.8% without any light-outcoupling enhancement. We attribute these high performances to the efficient TADF properties of the emitters with high PLQY and small Δ*E*_ST_. However, substantial efficiency roll-off is observed for all devices. This can be mainly attributed to the long delayed fluorescence lifetime of the emitters that may increase the probability of exciton quenching processes such as triplet-triplet annihilation (TTA) and triplet-polaron annihilation (TPA) in the devices. Interestingly, it is noted that the EQE values obtained in this study are somewhat different from the trend found in the PLQYs of the host films. Device **D1**, which is based on the less emissive DPAC*o*BA (**1**), exhibits higher EQEs than devices **D2** and **D3** although the PLQY of **1** is sufficiently high to afford the observed EQEs anyway; further optimization of the device structure would probably result in a more accurate trend in the device efficiency but is beyond the scope of this work. Nevertheless, the results obtained for the devices in this study suggest that the cyclic boryl groups function as good acceptors for TADF emitters, being capable of exhibiting narrow bandwidth emission and high device efficiency.

**Table 3:** Device performance of the TADF-OLEDs fabricated with **1–3**.

**Device (emitter)^**a**^**	**λ_**EL**_ (nm)**	**λ_**FWHM**_ (nm)^**b**^**	**CIE (x, y)^**c**^**	***V*_**on**_ (V)^**d**^**	**EQE (%)^**e**^**	**PE (lm W^**−1**^)^**f**^**	**CE (cd A^**−1**^)^**g**^**
**D1** (**1**)	464	70	(0.167, 0.191)	3.7	25.8	31.1	34.8
**D2** (**2**)	460	59	(0.151, 0.128)	3.6	16.2	15.0	17.7
**D3** (**3**)	492	83	(0.208, 0.421)	3.6	20.5	38.0	54.8

a *ITO (150 nm)/PEDOT:PSS (40 nm)/TAPC (20 nm)/mCP (10 nm)/DPEPO:emitter (20 wt%, 25 nm)/DPEPO (10 nm)/TPBi (30 nm)/LiF (1 nm)/Al (100 nm)*.

b *Full width at half maximum (FWHM)*.

c *Color coordinates (CIE 1931) at maximum luminance*.

d *V_on_: Applied voltage at a luminance of 1 cd m^-2^*.

e *Maximum external quantum efficiency*.

f *Maximum power efficiency*.

g *Maximum current efficiency*.

## Conclusion

We have demonstrated the impact of boron acceptors on the TADF properties of *ortho*-donor-appended triarylboron compounds, which consist of an *ortho* D–A backbone structure containing cyclic boryl (**1** and **2**) or BMes_2_ (**3**) groups as acceptors and a fixed DPAC donor. The compounds possessed a twisted structure and were sterically congested around the boron atom. All compounds showed strong TADF properties with high PLQYs in both solution and solid state. Blue-shifted fluorescence with narrower bandwidths was observed for the compounds bearing cyclic boryl acceptors (**1** and **2**) compared with that of the BMes_2_-containing **3**. TADF-OLEDs fabricated with **1**–**3** as emitters exhibited high device performance, and those based on the cyclic boryl emitters showed pure blue emission and narrower EL bands than the device with **3**. A high EQE of 25.8% was also achieved for the device fabricated with emitter **1**. The findings in this study suggest that the cyclic boryl groups may be useful for designing TADF emitters with narrow bandwidth emission and high device efficiency.

## Materials and Methods

### General Considerations

All operations were performed under an inert nitrogen atmosphere using standard Schlenk and glovebox techniques. Anhydrous grade solvents were dried by passing them through an activated alumina column and stored over activated molecular sieves (5 Å). Spectrophotometric-grade solvents for photophysical measurements were used as received. Commercial reagents were used without further purification after purchase. 2-Bromo-1-iodo-3-methylbenzene (Ikeuchi et al., 2019), 9-bromo-9,10-dihydro-9-boraanthracene (Zhou et al., 2012), and 10-bromo-9-oxa-10-boraanthracene (Melaïmi et al., 2006) were prepared according to the literature procedures. Deuterated solvents from Cambridge Isotope Laboratories were used. NMR spectra were recorded on a Bruker AM 300 (300.13 MHz for ^1^H, 75.48 MHz for ^13^C, and 96.29 MHz for ^11^B) spectrometer at ambient temperature. Chemical shifts are given in parts per million (ppm), and are referenced against external Me_4_Si (^1^H, ^13^C) and BF_3_·OEt_2_ (^11^B). Elemental analyses were performed on a Flash 2000 elemental analyzer (Thermo Scientific). Mass spectra were obtained using a JEOL JMS700 high-resolution EI-mass spectrometer (HR EI-MS) at the Korea Basic Science Institute, Daegu, Korea. Melting points (mp) were measured by Melting Point Apparatus SMP30 (Stuart Equipment). Thermogravimetric analysis (TGA) was performed with a TA Instruments Q50 under an N_2_ atmosphere at a heating rate of 20°C min^−1^. Cyclic voltammetry experiments were carried out using an Autolab/PGSTAT101 system.

### Synthesis of DPAC*o*Br

The mixture of 9,9-diphenyl-10*H*-acridine (0.56 g, 1.68 mmol), 2-bromo-1-iodo-3-methylbenzene (1.0 g, 3.37 mmol), tris-(dibenzylideneacetone)dipalladium(0) (Pd_2_(dba)_3_, 0.03 g, 0.02 mmol), tri-*tert*-butylphosphine (0.02 g, 0.06 mmol), and sodium *tert*-butoxide (0.32 g, 3.36 mmol) in dry toluene (20 mL) was refluxed for 48 h. After cooling down to room temperature, the mixture was diluted with CH_2_Cl_2_ (20 mL), filtered through a celite pad, and concentrated under reduced pressure. The crude product was purified by silica gel column chromatography using CH_2_Cl_2_/*n*-hexane (1:6, v/v) as an eluent to give 10-(2-bromophenyl)-9,9-diphenyl-10*H*-acridine (DPAC*o*Br) as a white solid (Yield: 0.68 g, 78%). ^1^H NMR (CD_2_Cl_2_): δ 7.43-7.36 (m, 2H), 7.26 (m, 6H), 7.09–6.99 (m, 7H), 6.94–6.87 (m, 4H), 6.29 (dd, *J* = 8.6, 0.6 Hz, 2H), 2.50 (s, 3H). ^13^C NMR (CD_2_Cl_2_): δ 147.2, 147.0, 141.4, 140.5, 139.2, 130.7, 130.6, 130.5, 130.4, 130.3, 128.81, 128.80, 128.4, 127.6, 127.5, 126.9, 126.2, 120.3, 113.4, 56.5, 23.6. HRMS (EI): m/z Calcd for C_32_H_24_BrN, 501.1092; Found, 501.1088.

### Synthesis of DPAC*o*BA (1)

To a solution of DPAC*o*Br (0.22 g, 0.44 mmol) in dry ether (30 mL) was added dropwise *n*-BuLi (0.18 mL, 0.44 mmol) at −30°C. The reaction mixture was allowed to warm to room temperature and stirred for 1 h. The mixture was cooled to −78°C and then 9-bromo-9,10-dihydro-9-boraanthracene (0.17 g, 0.67 mmol) in dry toluene (10 mL) was slowly added. After stirring at room temperature overnight, the mixture was concentrated under reduced pressure and purified by crystallization from CH_2_Cl_2_/MeOH to give DPAC*o*BA (**1**) as a white crystalline solid (Yield: 0.10 g, 37%). ^1^H NMR (CD_2_Cl_2_): δ 7.61 (t, *J* = 7.7 Hz, 1H), 7.47 (d, *J* = 7.5 Hz, 2H), 7.43–7.38 (m, 1H), 7.35 (td, *J* = 7.5, 1.5 Hz, 2H), 7.28 (d, *J* = 8.0 Hz, 1H), 7.22 (d, *J* = 7.8 Hz, 2H), 7.18–7.10 (m, 3H), 7.06–6.91 (m, 5H), 6.77–6.67 (m, 6H), 6.56 (ddd, *J* = 11.0, 8.2, 1.6 Hz, 4H), 6.01 (dd, *J* = 8.5, 1.1 Hz, 2H), 3.98 (d, *J* = 23.8 Hz, 1H), 3.65 (d, *J* = 23.7 Hz, 1H), 1.96 (s, 3H). ^13^C NMR (CD_2_Cl_2_): δ 149.1, 147.5, 144.5, 142.4, 142.3, 142.1, 137.7, 132.0, 131.2, 130.4, 129.1, 128.6, 128.5, 128.3, 127.7, 127.5, 127.1, 127.0, 126.01, 125.97, 125.4, 124.3, 119.5, 115.6, 56.0, 38.0, 22.0. ^11^B NMR (CD_2_Cl_2_): δ +58.2. Anal. Calcd for C_45_H_34_BN: C, 90.15; H, 5.72; N, 2.34%. Found: C, 90.03; H, 5.66; N, 2.58%. mp = 297°C. *T*_d5_ = 324°C.

### Synthesis of DPAC*o*OB (2)

This compound was analogously prepared following the procedures for **1** using DPAC*o*Br (0.30 g, 0.59 mmol), *n*-BuLi (0.24 mL, 0.60 mmol), and 10-bromo-9-oxa-10-boraanthracene (0.20 g, 0.78 mmol) to give DPAC*o*OB (**2**) as a white crystalline solid (Yield: 0.18 g, 50%). ^1^H NMR (CD_2_Cl_2_): δ 7.64 (t, *J* = 7.7 Hz, 1H), 7.56–7.49 (m, 2H), 7.46 (dd, *J* = 7.6, 1.4 Hz, 2H), 7.41 (d, *J* = 7.4 Hz, 1H), 7.36–7.26 (m, 3H), 7.19–7.10 (m, 3H), 7.06 (ddd, *J* = 8.4, 7.3, 1.6 Hz, 2H), 6.94–6.85 (m, 3H), 6.73 (m, 6H), 6.52 (dd, *J* = 8.0, 1.6 Hz, 2H), 6.46–6.38 (m, 2H), 5.80 (dd, *J* = 8.5, 1.2 Hz, 2H), 1.91 (s, 3H). ^13^C NMR (CD_2_Cl_2_): δ 158.5, 148.4, 143.8, 143.0, 142.4, 142.3, 136.0, 133.9, 131.2, 130.2, 129.4, 128.63, 128.58, 128.5, 127.2, 127.1, 126.9, 126.1, 125.7, 121.1, 119.5, 117.4, 115.2, 56.0, 22.2. ^11^B NMR (CD_2_Cl_2_): δ +64.8. Anal. Calcd for C_44_H_32_BNO: C, 87.85; H, 5.36; N, 2.33%. Found: C, 87.53; H, 5.28; N, 2.50%. mp = 325°C. *T*_d5_ = 333°C.

### Synthesis of DPAC*o*B (3)

This compound was analogously prepared following the procedures for **1** using DPAC*o*Br (0.30 g, 0.59 mmol), *n*-BuLi (0.24 mL, 0.60 mmol), and dimesitylboron fluoride (Mes_2_BF, 0.19 g, 0.72 mmol). Purification by silica gel column chromatography using CH_2_Cl_2_/*n*-hexane (1:6, v/v) as an eluent followed by recrystallization from CH_2_Cl_2_/MeOH afforded DPAC*o*B (**3**) as a green crystalline solid (Yield: 0.12 g, 31%). ^1^H NMR (CD_2_Cl_2_): δ 7.47 (t, *J* = 7.7 Hz, 1H), 7.33–7.22 (m, 4H), 7.15–7.11 (m, 5H), 6.96 (t, *J* = 7.4 Hz, 1H), 6.89 (d, *J* = 7.8 Hz, 1H), 6.80 (t, *J* = 7.2 Hz, 1H), 6.76–6.71 (m, 2H), 6.65 (d, *J* = 9.4 Hz, 6H), 6.31 (d, *J* = 8.2 Hz, 2H), 6.06 (d, *J* = 7.1 Hz, 1H), 5.69 (s, 1H), 2.20 (s, 3H), 2.18 (s, 3H), 2.15 (s, 3H), 2.01 (s, 3H), 1.93 (s, 3H), 1.81 (s, 3H), 0.62 (s, 3H). ^13^C NMR (CD_2_Cl_2_): δ 152.8, 148.1, 145.8, 143.3, 142.6, 141.9, 140.0, 138.7, 138.4, 137.2, 132.6, 131.7, 130.8, 129.9, 129.5, 128.0, 127.1, 126.3, 125.7, 125.4, 119.6, 118.8, 117.8, 117.2, 55.8, 26.0, 23.2, 22.6, 21.9, 20.6. ^11^B NMR (CD_2_Cl_2_): δ +84.0. Anal. Calcd for C_50_H_46_BN: C, 89.40; H, 6.90; N, 2.09%. Found: C, 89.05; H, 6.81; N, 2.28%. mp = 278°C. *T*_d5_ = 326°C.

### Cyclic Voltammetry

The redox behavior of compounds were examined by cyclic voltammetry measurements using a three-electrode cell configuration consisting of platinum working and counter electrodes and an Ag/AgNO_3_ (0.01 M in CH_3_CN) reference electrode. Oxidation curves were recorded in CH_2_Cl_2_ solutions (1 × 10^−3^ M), while reduction curves were obtained from THF (**2** and **3**) or DMSO (**1**) solutions (1 × 10^−3^ M). Tetra-*n*-butylammonium hexafluorophosphate (TBAPF_6_, 0.1 M) was used as the supporting electrolyte. The redox potentials were recorded at a scan rate of 100–200 mV s^−1^ and are reported against the Fc/Fc^+^ redox couple. The HOMO and LUMO energy levels were estimated from the electrochemical oxidation (*E*_1/2_) and reduction (E_onset_) peaks of cyclic voltammograms.

### Photophysical Measurements

UV/vis absorption and photoluminescence (PL) spectra were recorded on a Varian Cary 100 and FS5 spectrophotometer, respectively. Solution PL spectra were obtained from oxygen-free (N_2_-filled) and air-saturated toluene solutions in a sealed cuvette (typically 50 μM). PL spectra and PLQYs of doped host films were obtained on quartz plates. PLQYs of the samples were measured on an absolute PL quantum yield spectrophotometer (Quantaurus-QY C11347-11, Hamamatsu Photonics) equipped with a 3.3-inch integrating sphere. Transient PL decays were recorded on a FS5 spectrophotometer (Edinburgh Instruments) equipped with an OptistatDN^TM^ cryostat (Oxford Instruments).

### Fabrication of Electroluminescent Devices

OLED devices were fabricated on 25 × 25 mm glass substrate with half-patterned ITO layers (AMG). Glass substrates with pre-patterned ITO electrodes were cleaned by a sequential wet-cleaning processes in an ultrasonic bath (Song et al., 2018). After drying in a vacuum oven for a day, the substrates were subject to UV-plasma treatment for 1 min in a plasma cleaner (CUTE-MP, Femto Science). As a hole-injection layer, an aqueous dispersion of PEDOT:PSS (Clevios^TM^ P VP AI 4083, Heraeus) was spun (2,500 rpm for 30 s) onto the plasma-treated substrates and annealed on a hot plate (100°C for 10 min). Other organic and metal layers were sequentially deposited in a vacuum chamber (HS-1100, Digital Optics & Vacuum) at less than 1.5 × 10^−6^ torr. The current density–voltage–luminance (*J*–*V*–*L*) and angle-resolved electroluminescence (EL) intensity characteristics of the fabricated devices were obtained with a source-measure unit (Keithley 2400) using a calibrated photodiode (FDS100, Thorlab) and a fiber optic spectrometer (EPP2000, StellarNet) held on a motorized goniometer. The EQE (η_EQE_) and PE (η_PE_) of the devices were estimated from the measured full angular characteristics without Lambertian simplification. All device fabrication and measurement, except for the PEDOT:PSS coating, were carried out in a nitrogen (N_2_)-filled glove box, and all characteristics of the devices were measured at room temperature.

## Data Availability Statement

All datasets generated for this study are included in the article/Supplementary Material.

## Author Contributions

HM performed the synthesis and characterization. WL fabricated the devices. TL carried out DFT computation. JJ discussed the theoretical data. SY discussed the device data. ML designed the experiments and prepared the manuscript. All authors have approved the final version of the manuscript.

## Conflict of Interest

The authors declare that the research was conducted in the absence of any commercial or financial relationships that could be construed as a potential conflict of interest.

## Supplementary Material

These data include NMR spectra of compounds, crystallographic data, photophysical data, computational details, and electroluminescence results.
