# Supplementary material for: Impact of Boron Acceptors on the TADF Properties of Ortho-Donor-Appended Triarylboron Emitters
Source: Front Chem. 2020 Jun 24;8:538. doi: 10.3389/fchem.2020.00538 (PMC7344311; doi:10.3389/fchem.2020.00538)
Supplement: Supplementary file 1 [file Table_1.DOCX]

Supplementary Material

Impact of Boron Acceptors on the TADF Properties of *Ortho*-Donor-Appended Triarylboron Emitters

Hanif Mubarok,^1,¶^ Woochan Lee,^2,¶^ Taehwan Lee,^1^ Jaehoon Jung,^1^ Seunghyup Yoo^2^* and Min Hyung Lee^1^*

^1^Department of Chemistry, University of Ulsan, Ulsan 44610, Republic of Korea

^2^School of Electrical Engineering, KAIST, Daejeon 34141, Republic of Korea

syoo@ee.kaist.ac.kr (S.Y); lmh74@ulsan.ac.kr (M.H.L)

**Supplementary Figure 1.** ^1^H (bottom) and ^13^C (top) NMR spectra of DPAC*o*Br in CD_2_Cl_2_ († from residual CH_2_Cl_2_).

**Supplementary Figure 2.** ^1^H (bottom), ^13^C (middle), and ^11^B (top) NMR spectra of DPAC*o*BA (**1**) in CD_2_Cl_2_ († from residual CH_2_Cl_2_ and * from H_2_O).

**Supplementary Figure 3.** ^1^H (bottom), ^13^C (middle), and ^11^B (top) NMR spectra of DPAC*o*OB (**2**) in CD_2_Cl_2_ († from residual CH_2_Cl_2_ and * from H_2_O).

**Supplementary Figure 4.** ^1^H (bottom), ^13^C (middle), and ^11^B (top) NMR spectra of DPAC*o*B (**3**) in CD_2_Cl_2_ († from residual CH_2_Cl_2_ and * from H_2_O).

**X-ray Crystallography.** Single crystals of suitable size and quality (DPAC*o*OB, **2**) were coated with Paratone oil and mounted onto a glass capillary. Diffractrion data were obtained at 296 K. The crystallographic measurements were performed on a Bruker SMART Apex II CCD area detector diffractometer with a graphite-monochromated Mo-K*α* radiation (*λ* = 0.71073 Å). The structures were solved by direct methods^1^ and refined by full-matrix least-squares fitting on *F*^2^ using SHELXL-2014.^2^ All non-hydrogen atoms were refined with anisotropic displacement parameters. Hydrogen atoms were placed at their geometrically calculated positions and were refined riding on the corresponding carbon atoms with isotropic thermal parameters. Full details of the structure determinations have been deposited as a cif with the Cambridge Crystallographic Data Centre under the CCDC deposition number 1968375 (**2**). The data can be obtained free of charge via www.ccdc.cam.ac.uk.

**Supplementary Table 1**. Crystallographic data and parameters for **2**.

|  | **2** (DPAC*o*OB) |
| --- | --- |
| formula | C_44_H_32_BNO |
| formula weight | 601.51 |
| crystal system | Triclinic |
| space group | *P*−1 |
| *a* (Å) | 9.1417(2) |
| *b* (Å) | 9.5796(2) |
| *c* (Å) | 19.4378(4) |
| *α* (°) | 78.8745(12) |
| β (°) | 79.0187(12) |
| *γ* (°) | 74.1334(10) |
| *V* (Å^3^) | 1589.42(6) |
| Z | 2 |
| *ρ*_calc_ (g cm^−3^) | 1.257 |
| *μ* (mm^−1^) | 0.074 |
| *F*(000) | 632 |
| *T* (K) | 296(2) |
| *hkl* range | –11→11, –11→11, –23→23 |
| measd reflns | 20775 |
| unique reflns [*R*_int_] | 5831(0.0297) |
| reflns used for refinement | 5831 |
| refined parameters | 425 |
| R1*^a^* (I > 2σ(I)) | 0.0405 |
| wR2*^b^* all data | 0.1071 |
| GOF on *F*^2^ | 1.034 |
| *ρ*_fin_ (max/min) (e Å^−3^) | 0.176/−0.211 |

*^a^* R1 = ∑||*F*o| − |*F*c||/∑|*F*o|. *^b^* wR2 = {[∑*w*(*F*o^2^ − *F*c^2^)^2^]/[∑*w*(*F*o^2^)^2^]}^1/2^.

**Supplementary Figure 5.** Cyclic voltammograms of **1**−**3** in solution (1 × 10^−3^ M). Conditions: for oxidation, solvent: CH_2_Cl_2_, scan rate: 100 mV/s; for reduction, solvent: DMSO for **1** and THF for **2** and **3**, scan rate: 200 mV/s for **1** and 100 mV/s for **2** and **3**.

**Supplementary Table 2.** Electrochemical data for **1**−**3**.

| Compound | Oxidation (V)^a^ | Reduction (V)^b^ | *E*_g_ (eV) |
| --- | --- | --- | --- |
| **1** (DPAC*o*BA) | 0.51 | −2.19 | 2.70 |
| **2** (DPAC*o*OB) | 0.54 | −2.30 | 2.84 |
| **3** (DPAC*o*B) | 0.54 | −2.28 | 2.82 |

^a^Half-wave potential (*E*_1/2_). ^b^Onset potential (*E*_onset_).

**Supplementary Figure 6.** (Left) PL spectra of **1**−**3** in oxygen-free and air-saturated toluene at 298 K. (Right) Transient PL decay curves of **1**−**3** in oxygen-free toluene at 298 K. Inset: PL decay curves in oxygen-free and air-saturated toluene.

**Supplementary Figure 7.** Fluorescence and phosphorescence spectra of **1**−**3** in toluene at 77 K.

**Supplementary Figure 8.** PL spectra and transient PL decay curves of the DPEPO host films doped with 20 wt% of **1**−**3**. Inset: photos of the films under UV lamp.

**Supplementary Figure 9.** TGA curves of **1**−**3**.

**Theoretical Calculations.** The computational study based on the density functional theory (DFT) was carried out to elucidate the geometrical and photophyscial properties. The PBE0 hybrid functional^3^ and 6-31G(d,p) basis set implemented in GAUSSIAN 16 software package^4^ were used. The ground (S_0_) states of compounds were optimized using DFT calculations, and their lowest singlet (S_1_) and triplet (T_1_) excited states were optimized using time-dependent DFT (TD-DFT) calculations with the same functional and basis set. The polarizable continuum model using the integral equation formalism (IEFPCM) was employed to take account for the influence of solvent medium (toluene).^5^ The composition of frontier molecular orbital and the overlap integral extents were computed using AOMix^6^ and Multiwfn^7^ programs, respectively.

**Supplementary Table 3.** The MO energy (eV) and contribution (in %) of donor and acceptor moieties to the frontier molecular orbitals and overlap integrals (*I*_H/L_) between them for the groun state geometries of **1**−**3**. The energy of the S_1_ state relative to the S_0_ state is provided.

|  | MO | energy  (eV) | donor  (DPAC) | acceptor | | *I*_H/L_ | Δ*E*(S_1_-S_0_) |
| --- | --- | --- | --- | --- | --- | --- | --- |
|  |  |  |  | MePh | BAr_2_ | (%) | (eV) |
| **1** (DPAC*o*BA) | LUMO | −1.54 | 1.46 | 2.38 | 96.2 | 19.6 | 2.671 |
|  | HOMO | −5.37 | 82.9 | 2.93 | 14.2 |  |  |
| **2** (DPAC*o*OB) | LUMO | −1.56 | 0.91 | 1.71 | 97.4 | 18.0 | 2.797 |
|  | HOMO | −5.40 | 83.1 | 2.89 | 14.1 |  |  |
| **3** (DPAC*o*B) | LUMO | −1.59 | 1.03 | 18.7 | 80.3 | 18.1 | 2.640 |
|  | HOMO | −5.44 | 89.8 | 3.04 | 7.16 |  |  |

**Supplementary Table 4.** The calculated absorption wavelength (*λ*_abs_, in nm) and the corresponding oscillator strength (*f*) for **1**−**3**.

|  | *λ*_abs_ | *f*_abs_ | Major contribution |
| --- | --- | --- | --- |
| **1** (DPAC*o*BA) | 404 | 0.0285 | HOMO → LUMO (99.6%) |
| **2** (DPAC*o*OB) | 401 | 0.0266 | HOMO → LUMO (99.6%) |
| **3** (DPAC*o*B) | 411 | 0.0046 | HOMO → LUMO (98.8%) |

**Supplementary Table 5.** The calculated emission wavelength (*λ*_em_, in nm) and the corresponding oscillator strength (*f*) for **1**−**3**.

|  | *λ*_em_ | *f*_em_ | Major contribution |
| --- | --- | --- | --- |
| **1** (DPAC*o*BA) | 530 | 0.0009 | HOMO → LUMO (99.7%) |
| **2** (DPAC*o*OB) | 506 | 0.0045 | HOMO → LUMO (99.7%) |
| **3** (DPAC*o*B) | 531 | 0.0014 | HOMO → LUMO (99.0%) |

**Supplementary Figure 10.** Angular spectral dependence of the fabricated TADF-OLEDs with the emitters, (a) **1** (DPAC*o*BA), (b) **2** (DPAC*o*OB), and (c) **3** (DPAC*o*B).

**Supplementary Figure 11.** (a) Power efficiency-luminance (*η*_PE_−*L*) characteristics and (b) Current efficiency-current density (*η*_CE_−*J*) characteristics of devices (**D1**−**D3**).

**Supplementary References**

1. Sheldrick, G. M. *SHELXS-97: Program for the Solution of Crystal Structures*, University of Göttingen, Germany, 2008.

2. Sheldrick, G. M. *Acta Crystallogr. A* **2008**, *64*, 112-122.

3. Adamo, C.; Barone, V. *J. Chem. Phys.* **1999**, *110*, 6158-6170.

4. Frisch, M. J.; Trucks, G. W.; Schlegel, H. B.; Scuseria, G. E.; Robb, M. A.; Cheeseman, J. R.; Scalmani, G.; Barone, V.; Petersson, G. A.; Nakatsuji, H.; Li, X.; Caricato, M.; Marenich, A. V.; Bloino, J.; Janesko, B. G.; Gomperts, R.; Mennucci, B.; Hratchian, H. P.; Ortiz, J. V.; Izmaylov, A. F.; Sonnenberg, J. L.; Williams-Young, D.; Ding, F.; Lipparini, F.; Egidi, F.; Goings, J.; Peng, B.; Petrone, A.; Henderson, T.; Ranasinghe, D.; Zakrzewski, V. G.; Gao, J.; Rega, N.; Zheng, G.; Liang, W.; Hada, M.; Ehara, M.; Toyota, K.; Fukuda, R.; Hasegawa, J.; Ishida, M.; Nakajima, T.; Honda, Y.; Kitao, O.; Nakai, H.; Vreven, T.; Throssell, K.; Montgomery Jr., J. A.; Peralta, J. E.; Ogliaro, F.; Bearpark, M. J.; Heyd, J. J.; Brothers, E. N.; Kudin, K. N.; Staroverov, V. N.; Keith, T. A.; Kobayashi, R.; Normand, J.; Raghavachari, K.; Rendell, A. P.; Burant, J. C.; Iyengar, S. S.; Tomasi, J.; Cossi, M.; Millam, J. M.; Klene, M.; Adamo, C.; Cammi, R.; Ochterski, J. W.; Martin, R. L.; Morokuma, K.; Farkas, O.; Foresman, J. B.; Fox, D. J. *Gaussian 16, Revision A.03*, Gaussian, Inc., Wallingford CT, 2016.

5. Tomasi, J.; Mennucci, B.; Cammi, R. *Chem. Rev.* **2005**, *105*, 2999-3094.

6. Gorelsky, S. I. AOMix: Program for Molecular Orbital Analysis, version 6.94, 2018. http://www.sg-chem.net.

7. Lu, T.; Chen, F. *J. Comput. Chem.* **2012**, *33*, 580-592.
